# An Assessment of Fixed Interval Timing in Free-Flying Honey Bees (*Apis mellifera ligustica*): An Analysis of Individual Performance

**DOI:** 10.1371/journal.pone.0101262

**Published:** 2014-07-01

**Authors:** David Philip Arthur Craig, Christopher A. Varnon, Michel B. C. Sokolowski, Harrington Wells, Charles I. Abramson

**Affiliations:** 1 Department of Psychology, Oklahoma State University, Stillwater, Oklahoma, United States of America; 2 Department of Psychology, Université de Picardie Jules Verne, Amiens, Picardy, France; 3 Department of Biology, University of Tulsa, Tulsa, Oklahoma, United States of America; Monash University, Australia

## Abstract

Interval timing is a key element of foraging theory, models of predator avoidance, and competitive interactions. Although interval timing is well documented in vertebrate species, it is virtually unstudied in invertebrates. In the present experiment, we used free-flying honey bees (*Apis mellifera ligustica*) as a model for timing behaviors. Subjects were trained to enter a hole in an automated artificial flower to receive a nectar reinforcer (i.e. reward). Responses were continuously reinforced prior to exposure to either a fixed interval (FI) 15-sec, FI 30-sec, FI 60-sec, or FI 120-sec reinforcement schedule. We measured response rate and post-reinforcement pause within each fixed interval trial between reinforcers. Honey bees responded at higher frequencies earlier in the fixed interval suggesting subject responding did not come under traditional forms of temporal control. Response rates were lower during FI conditions compared to performance on continuous reinforcement schedules, and responding was more resistant to extinction when previously reinforced on FI schedules. However, no “scalloped” or “break-and-run” patterns of group or individual responses reinforced on FI schedules were observed; no traditional evidence of temporal control was found. Finally, longer FI schedules eventually caused all subjects to cease returning to the operant chamber indicating subjects did not tolerate the longer FI schedules.

## Introduction

A sense of time appears to be of fundamental importance for animals and can be observed in the ubiquity of circadian rhythms in species of the animal kingdom. Moreover, circadian rhythms have been linked to behavioral and physiological adaptation involving both biotic and abiotic environmental factors [1]. The molecular underpinnings for circadian rhythms appear to have arisen more than once in the early evolution of life [2]; circadian rhythms have been shown to increase fitness in organisms as simple as cyanobacteria [3], [4].

Perception of time intervals that are shorter than circadian rhythms is also important for animal fitness. Interval timing is central in foraging theory wherein maximizing caloric gain over a period of time is expected to increase fitness [5]–[7]. Furthermore, many aspects of escaping predators depend on interval timing (e.g. evasive moves are unsuccessful if they occur too early or too late) [8]. Finally, competitive interactions involving reproduction may also rely on interval timing for some species when communication is involved [9].

However, a distinction between circadian rhythms and arbitrary interval timing has been made [8]; circadian rhythms are a form of “timing”, but investigating circadian rhythms does not necessarily assess adaptability or learning in an individual organism. The present investigation's findings do not necessarily generalize to the natural rhythms of insects [10]; instead, we focus on the ability of honey bees to adapt to and perform on arbitrary time intervals. Fixed interval (FI) schedules are one of the most basic assessments of arbitrary interval timing and have traditionally been observed to produce qualitative “scalloped” or “break-and-run” cumulative response curves in organisms as well as lower session response rates compared to responding on continuous reinforcement (CRF) [11]–[17].

Following B.F. Skinner's example, an assumption that all organisms produce similar patterns became firmly rooted in early behavioral investigations. Early comparative assessments demonstrated qualitative differences in response performance via phyletic comparisons [18]; a variety of species have now been investigated and compared on FI schedules to better assess the generality of the properties of timing across species. While many vertebrate species have been investigated for interval timing abilities [19], [20], only two FI timing investigations have been conducted using invertebrates as model organisms; both studies produced seemingly contrasting findings [21], [22].

[22] placed bumble bees' (*Bombus impatiens*) proboscis extension responding in an artificial flower on various short FI schedules or a long FI schedule (either FI 6-sec or FI 12-sec in one condition and an FI 36-sec in a second condition) or on multiple timing schedules (either FI 6-sec or FI 12-sec with the FI 36-sec schedule intermixed in the same condition). While reviewing [22], [23] maintained that bumble bees do not differ from vertebrates in their responding on FI schedules. We believe this conclusion of timing was premature because [22] only compared group averages of post-reinforcement pause (PRP) between FI schedule groups and did not include measures of response patterns or response rate. Multiple measures are beneficial for timing investigations as changes in PRP do not necessarily imply temporal control [24], for significant PRP change yields relatively little information about responding.

In contrast to bumble bees, [21] observed no evidence of temporal control in honey bee (*Apis mellifera*) cumulative curves as no “scallops” or “break-and-run” response patterns were observed when hole-entering responding was reinforced on FI 6-sec, FI 9-sec, FI 12-sec, FI 15-sec, and FI 20-sec schedules. [21] offered visual inspections of cumulative curves but did not measure interresponse time, so PRP and response rate could not be analyzed quantitatively. However, the lack of a noticeable “scallop” or “break-and-run” cumulative curve pattern implies the lack of both a noticeable PRP and an accelerating response rate as reinforcement availability approaches.

For honey bees and bumble bees, the floral landscape changes during each day from the endogenous patterns of nectar secretion and pollen presentation of angiosperm species [25]–[27]. Clearly, honey bee colonies shift their foraging among food sources within each day to match the temporal productivity of different floral resources [28], [29]. Also, honey bees visiting a flower patch are able to learn to maximize net energy gain through decisions that increase caloric consumption [30]–[36], reduce flight time between flowers [37], [38], and minimize flower handling time [39], [40]. These findings may imply some form of interval timing by honey bees. Nevertheless, in contrast to bumble bees, [21] concluded that there was no evidence of interval timing in honey bees.

While these comparative findings could indicate invertebrate differences, no replications have been conducted in the investigated species. Additionally, the development of modern methods to record PRP and response rate as well as modern quantitative data analysis methods (i.e. null hypothesis significance testing) may be why modern investigations have claimed evidence of temporal control. Indeed, [20] report individual fresh water turtles did not display convincing temporal control [24], but aggregating the individuals' data produced evidence of temporal control. These types of unrepresentative aggregates may have been reported in [22]. To address these issues, we utilized a honey bee invertebrate model and analyzed non-aggregated representations of PRP and response rate by assessing our individuals' data with Observation Oriented Modeling [41]. We analysed response rate changes within fixed interval trials, compared baseline PRPs with PRPs occurring during the fixed interval sessions, and compared performances between the first and final fixed interval session. This individual analysis is a unique contribution to the temporal control literature.

## Methods

### Subjects

Subjects were wild free-flying *Apis mellifera* L. (n = 50) from the Oklahoma State University Comparative Psychology and Behavioral Biology Laboratory apiary. Free-flying subjects were utilized to increase ecological validity. During the experiment, subjects flew from their hive to feed in an operant chamber. Subjects also had constant access to one 10% sucrose solution feeding station near the operant chamber. As Oklahoma State University does not require an ethics institutional review for non-threatened invertebrates, no specific permits were required to conduct the present study. All subjects were experimentally naïve prior to the experiment.

### Apparatus

We concurrently utilized two adjoined computer-controlled clear acrylic operant chambers (24 cm×26 cm×38 cm) that provided 50% sucrose solution [42]. The operant chambers were located approximately 3 m from the 10% sucrose solution feeding station. The top of an operant chamber served as a door the experimenter opened and closed once the subject attempted to enter or leave the apparatus. Subjects attempting to enter the apparatus flew in circles above the top of the operant chamber and subjects attempting to leave the apparatus flew inside of the operant chamber directly below the top of the operant chamber. Once inside the operant chamber, subjects orientated themselves towards the response hole (diameter: 5 mm) located in the center of the side of the apparatus opposite of the adjoining wall separating each operant chamber. A response was recorded when the subject entered the response hole in the operant chamber and broke an infrared beam located 1 cm within the response hole. The response was considered complete when the subject exited the response hole. Thus, to make multiple responses, the subject was required to repeatedly enter and exit the response hole. When reinforcement contingencies were met, 5µl of 50% sucrose solution was released via a computer-controlled stepper motor into a cup attached to the end of the response hole located in front of the subject's head while she was still inside the response hole. The stepper motor served as a consistent marking stimulus, for the motor lightly sounded and vibrated the apparatus upon reinforcement delivery. A full explanation of the apparatus and calibration data is available in [43].

### Shaping

Subjects were randomly collected from the 10% sucrose solution feeding station and were brought to the operant chamber where hole-entering responses were shaped. During shaping, drops of sucrose solution were placed near the response hole and then inside the response hole. Some subjects quickly learned to enter the response hole after being placed in the operant chamber while others needed to be placed directly in the response hole before learning to enter the hole for sucrose reinforcement. Shaping was considered complete once the subject consistently returned to the operant chamber directly from the hive. After subjects were trained to make the response, the newly trained subjects were able to recruit additional potential subjects.

After shaping, each subject was tagged so the subjects could be distinguished. We used a Queen Marking Tube (QMT1) to immobilize the subject while a colored, numbered tag was attached with a non-toxic adhesive; these materials were purchased from Betterbee (Greenwich, NY). We attempted to minimize the duration the subject was restrained to reduce subject stress; we also provided the subject with three drops of 50% sucrose solution after tagging to try to counteract any punishing effect of the tagging procedure.

### Sessions

We utilized the cyclical foraging patterns of our free-flying honey bees to separate sessions; we collected all session data for each subject in a single day. Each visit to the apparatus after returning from the hive was considered a separate session. Throughout the experiment, a session was initiated by a subject's first response in the operant chamber after returning from the hive. Each session ended as the subject completed its final response prior to returning to the hive; we waited until the subject returned to the hive before considering a session complete. As each session's duration was determined by the subject's behavior, session duration were not identical. In addition to variable session durations, we did not control the number of trials per session. Honey bees can hold between 50 µl to 80µl of solution and return to the hive to unload after filling their social crop; hence, each session could offer anywhere between 10 to 16 reinforcers. This variability in the number of reinforces per session is an inherent aspect of working with unconfined and wild subjects in a naturalistic setting.

If a subject left the operant chamber during a session, we visually followed the subject to determine if she returned to the hive or the nearby 10% sucrose solution feeding station. If the subject returned to the hive, the session was considered complete, and another session began when the subject returned to the operant chamber. However, if the subject returned to the 10% sucrose solution feeding station and extended its proboscis or did not return to the operant chamber after 30 minutes, data collection was terminated for that subject.

Sessions began after hole-entering responding was shaped and subjects directly returned to the operant chamber after leaving the hive. We collected data from the first two bees that visited the apparatus for 27 sessions or until the subject stopped returning to the apparatus. All subjects completed the 27 sessions in one day; we could not collect data over sequential days and were thus obliged to limit our number of sessions. We did not collect data over multiple days because we were unable to confine our subjects to assure subjects were not foraging at different locations and thus experiencing different reinforcement contingencies between days. However, we were able to ensure subjects were only foraging at the operant chamber throughout the experiment, for we visually followed subjects to be sure they returned to the operant chamber immediately after leaving the hive.

We recorded responses per session, response duration, reinforcers per session, interresponse time (IRT), and intersession intervals while also recording environmental temperature. Adding response duration and IRT intervals together for each session produced session duration and dividing this value by the number of responses made during a session produced an average response rate per session for each bee.

### Baseline

Six baseline sessions of continuous reinforcement (CRF) were administered so that each bee could serve as her own control. Previous investigations [42] have revealed honey bees begin consistently responding in the utilized operant chamber after approximately five CRF sessions. During baseline sessions, subjects were allowed to freely enter the operant chamber, respond, and exit the operant chamber to avoid potential post-reinforcement delay effects [42].

### Fixed Interval Schedules

After six sessions of baseline CRF were completed, subjects entered the experimental condition for 20 sessions wherein responding was reinforced on either an FI 0-sec (CRF), FI 15-sec, FI 30-sec, FI 60-sec, or FI 120-sec schedule of reinforcement. We selected these intervals to remain consistent with the intervals used in [44], [45]; however, we added a shorter FI 15-sec schedule as utilizing a greater number of schedules is recommended [46]. We did not select intervals shorter than 15-sec because previous investigations [42] revealed responding reinforced on CRF schedules produce IRTs ranging between one to ten seconds; responding would likely not come under temporal control under a short FI schedule that is similar in duration to a typical IRT under CRF contingencies.

The first response of each new session was reinforced and we initiated the fixed interval when the subject exited the response hole to offer a fixed temporal reference for the subject. This protocol differs from traditional FI investigations [12] wherein the first response of a new session was reinforced on an FI schedule rather than a CRF schedule. We were obliged to utilize the present protocol because we observed pilot subjects attempted to leave the operant chamber if, upon entering the apparatus, more than approximately ten responses were made without reinforcement. Without reinforcing the first response of each session, we would have been unable to administer the relatively long schedules we sought to investigate. Once the schedule was initiated, subjects were free to respond throughout the fixed interval and these responses did not reset the interval.

### Extinction

Following the experimental FI condition (sessions 7–26), a single 10-min extinction session was administered during the 27^th^ session to determine if the varying fixed interval schedules produced changes in extinction responding [47].

### Groups

Subjects were randomly assigned to five groups of differing FI schedules with 10 subjects in each group. For all groups, responding was reinforced continuously for the first six sessions so that each individual's and group's baseline performances could be compared to responding on the FI schedules. Following the six baseline sessions, 20 FI sessions were administered; FI schedule duration served as the only manipulated difference between groups. The groups were named according to the conditions and FI schedule to which subjects were assigned and serve to indicate the utilized ABC repeated measures design: 0-0-X, 0-15-X, 0-30-X, 0-60-X, and 0-120-X. The first number represents the CRF baseline (an FI 0-sec schedule), the second number represents the FI schedule of the experimental condition (i.e. the group assignment), and the X represents extinction.

### Data Analysis

We used Observation Oriented Modeling [41], [48] which is a data analysis technique that permitted us to compare our observed results to expected patterns of outcomes for each bee and then to evaluate the differences with an accuracy index and a randomization test. Observation Oriented Modeling (OOM) assesses individual subject observations and does not rely on traditional summaries of data such as measures of central tendency or variability. By using these methods, we were able to eschew the assumptions of null hypothesis significance testing (e.g., homogeneity, normality) as well as avoid construing learning as an abstract population parameter such as a mean or variance to be estimated from our data.

Within OOM, we performed a series of ordinal analyses which produce a proportion correct classification (PCC) value and a chance-value (a probability statistic). For each analysis, an observed PCC value was computed by comparing an *a priori* ordinal prediction with direct pair-wise comparisons of the observed data. The resulting PCC value ranges from 0 to 1 and is the proportion of the observed data that matches the expected pattern. Higher values indicate more observations were correctly classified by the prediction.

Next, a randomization process wherein the observed data were randomly shuffled between groups/conditions was repeated 100 times for each ordinal analysis to create a range of randomized PCC values. The observed PCC values were then compared to the randomized range of PCC values to compute a chance value (*c*-value). The *c*-value ranges from 0 to 1 and displays the proportion of randomized versions of the observed data that yielded PCC values greater than or equal to the observed data's PCC value. For example, a *c*-value of.01 indicates the observed PCC value was larger than 99 of the PCC values obtained from 100 randomized versions of the data. In a two-order comparison, a *c*-value could be considered as conceptually similar to a binomial probability. However, as *c*-values are calculated from randomizations of the observed data points, each PCC value is assessed on an adaptable distribution that is based on observed data rather than a hypothetical distribution (e.g. the standard normal curve). [49] thoroughly describes numerous philosophical and practical differences between Observational Oriented Modeling versus null hypothesis significance testing and contains and compares data sets analyzed via both methods. For a summary of randomization tests, see [50].

For the present data, we were unable to perform null hypothesis significance testing (NHST) for numerous reasons. First, many subjects dropped-out while other subjects did not; subjects that ceased returning to the operant chamber after 30 minutes or foraged outside of the operant chamber were considered drop-outs. Thus, no conceptual population parameter can be assessed, for multiple patterns of responding emerged within groups. Second, because of these drop-outs, using NHST would prevent analysis of over half of our subjects' data; we were able to analyze all the collected data using OOM. Third, our data do not meet homogeneity assumptions. Fourth, our data do not meet normality assumptions required to abstract to a population's presumed normal curve. Finally, the number of pairwise comparisons we explored would have depleted our alpha levels and power; however, because we did not generalize the data to a normal distribution, alpha levels, degrees of freedom, and power are of no concern.

### Procedural Differences

Two procedural differences between the literature and present experiment complicate a comparison with previous invertebrate FI investigations. First, we measured a different response (completely entering a response hole) compared to the head entering response reported in [21] and proboscis extension response reported in [22]. Second, we used a response-initiated protocol similar to [51] wherein that the first response of each session was immediately reinforced to decrease drop-out rates. Pilot investigations revealed subjects leave the apparatus if reinforcement was not provided after as few as 10 unreinforced responses were made; in order to collect timing data, we had to reinforce the first response of each session. Honey bees typically survey multiple foraging locations but will return to a single location if sucrose is consistently present; reinforcing the first response of a session signals sucrose is still available at this foraging location after the subject returns from the hive. Thus, reinforcing the first response of a visit to the operant chamber serves as the motivating operation of the protocol, for without this first response, the subject will cease responding and drop-out of the experiment by surveying other foraging locations (e.g. 10% sucrose feeder, flowers).

The reason the present protocol differs from previous FI investigations is because the present experiment differs in its goals compared to many previous FI investigations. Previously, only two behavioral investigations of timing in bees exist and we believe temporal control (or any behavioral phenomena) should first be demonstrated in a simple protocol before using more complicated methods [23]. As such, we simplified our protocol compared to previous invertebrate temporal control investigations in three ways.

First, we sought to investigate the immediate shift from CRF to relatively long FI schedules to determine which intervals are able to immediately maintain long-term responding. We did not adhere to the method of increasing the duration of our FI schedules for individual subjects in an attempt to maintain responding as reported in [21]; we instead utilized a protocol similar to [45]. Immediately changing the response contingencies from CRF to a single FI schedule is a more pure assessment of timing, for each subsequent FI schedule change may be contaminated by a previous FI schedule. Additionally, increasing the duration of a fixed interval within subjects could result in an assessment of persistence and may not address the simple timing process of using a single schedule of FI for each subject. Finally, as the number of delivered reinforcers varies within sessions and between subjects, an effective and simple comparison using incremental FI schedules would be impossible.

Second, we did not use compound schedules like [22] to demonstrate timing for three reasons. First, utilizing more complicated schedules assumes temporal control can occur on simple FI schedules of reinforcement; however, [21] did not find evidence of temporal control in honey bees using a comparatively simpler process of single FI schedules; thus, this assumption has not been supported. Second, some of the earliest compound schedules were developed to demonstrate a variety of subtle drug effects on temporal control that otherwise simpler FI schedules would not illuminate [52], [53]. We did not perform a drug manipulation, nor were we interested in teasing apart the subtleties of behavior that has yet to be established. Third, many compound schedules may contaminate responding and stifle behaviors coming under temporal control. In short, we believe assessing temporal control in a simple schedule protocol should precede investigations of compound FI schedules.

Third, temporal investigations have incorporated on/off cues that signal when reinforcement is available into classical FI protocols. We did not use this method as the addition of a signal may turn the schedule into a go-no/go protocol and could result in a discrimination assessment rather than the intended timing investigation. This procedure is fundamentally different than the signaling of reinforcement-availability after the contingency has been met to indicate the duration reinforcement is available [54]. We believe fixed interval investigations utilizing signals before the contingency has been met do not assess temporal control of primary reinforcement availability.

## Results

**Figure 1:**
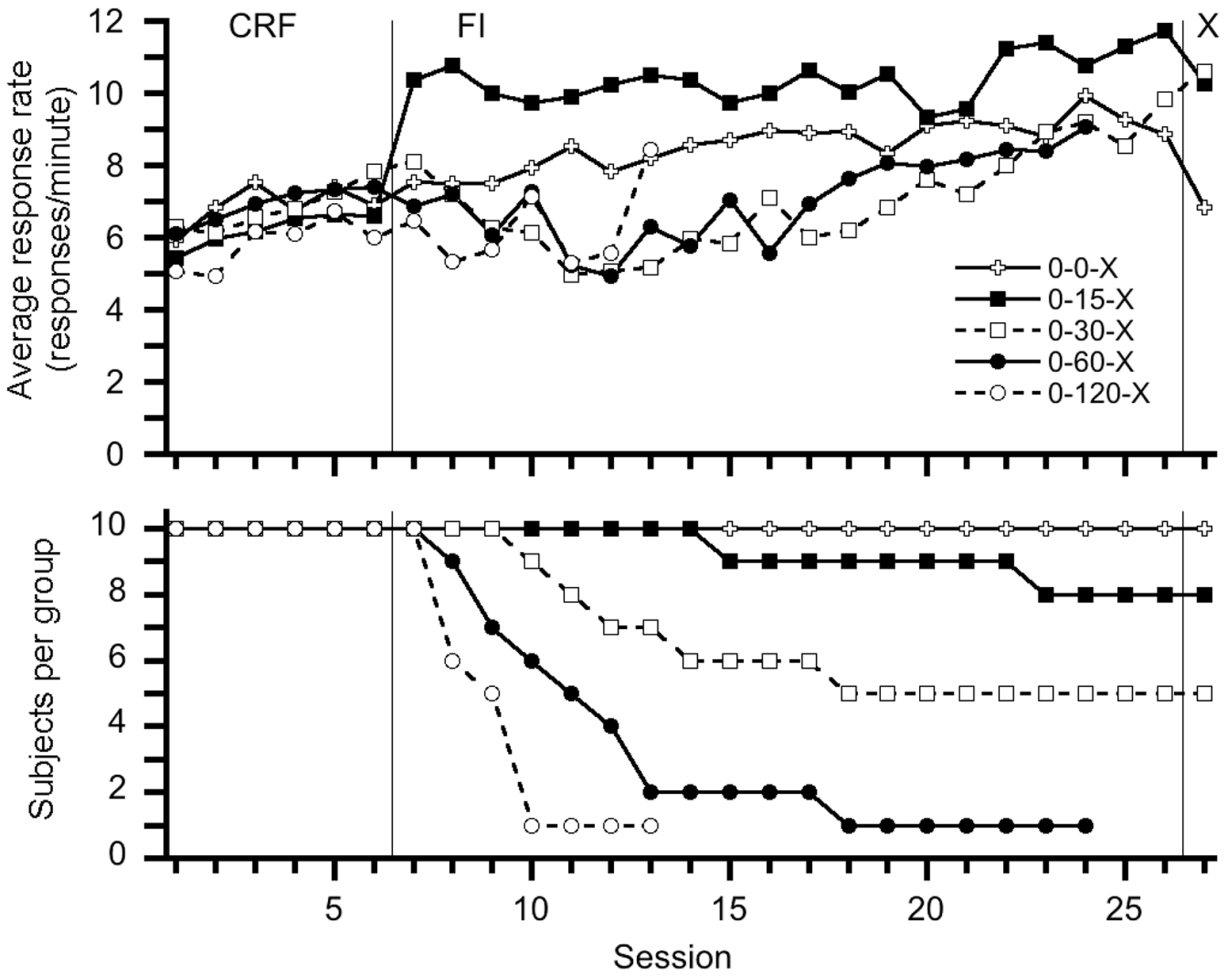
Group average response rates for each session and a depiction of what session subjects ceased responding. A clear increase in response rate is observed, on average, as the fixed interval sessions continue throughout the experiment.

**Figure 2:**
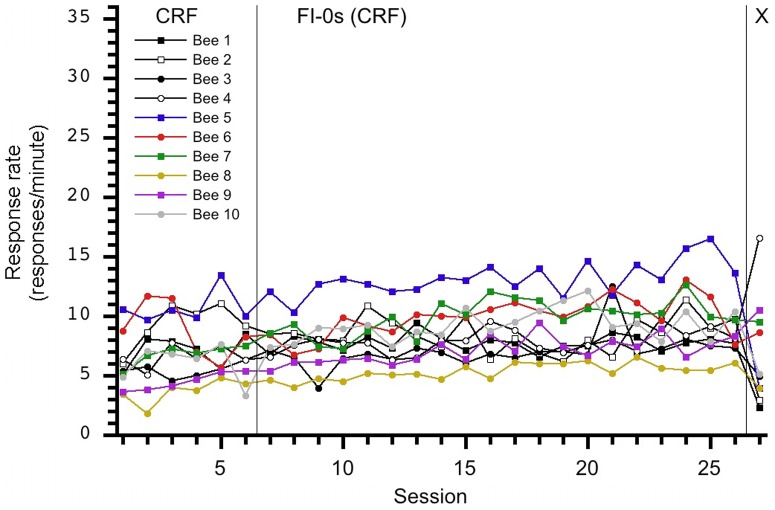
Individual response rates per session for the control, 0-0-X, group. A slight, general increase in response rate is observed for most subjects as CRF sessions continue.

**Figure 3:**
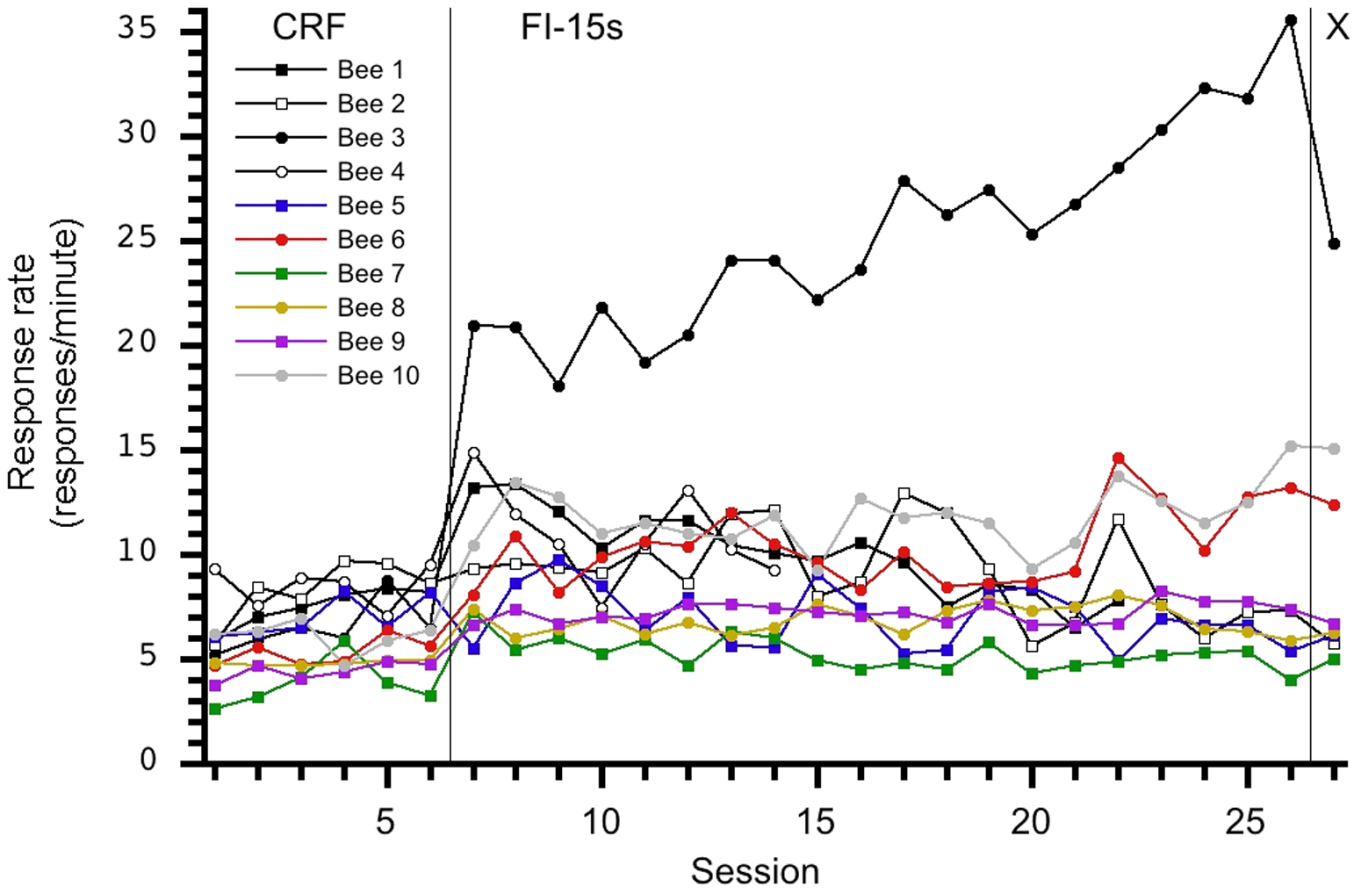
Individual response rates per session for the 0-15-X group. Bee 3′s response pattern clearly differs from the other nine subjects; this relatively high response rate affected the group average performance as depicted in Figure 1. A sudden, yet small, increase in response rate is observed during the transition from CRF to FI-15s, and responding was maintained at a relatively uniform response rate on the FI schedule.

**Figure 4:**
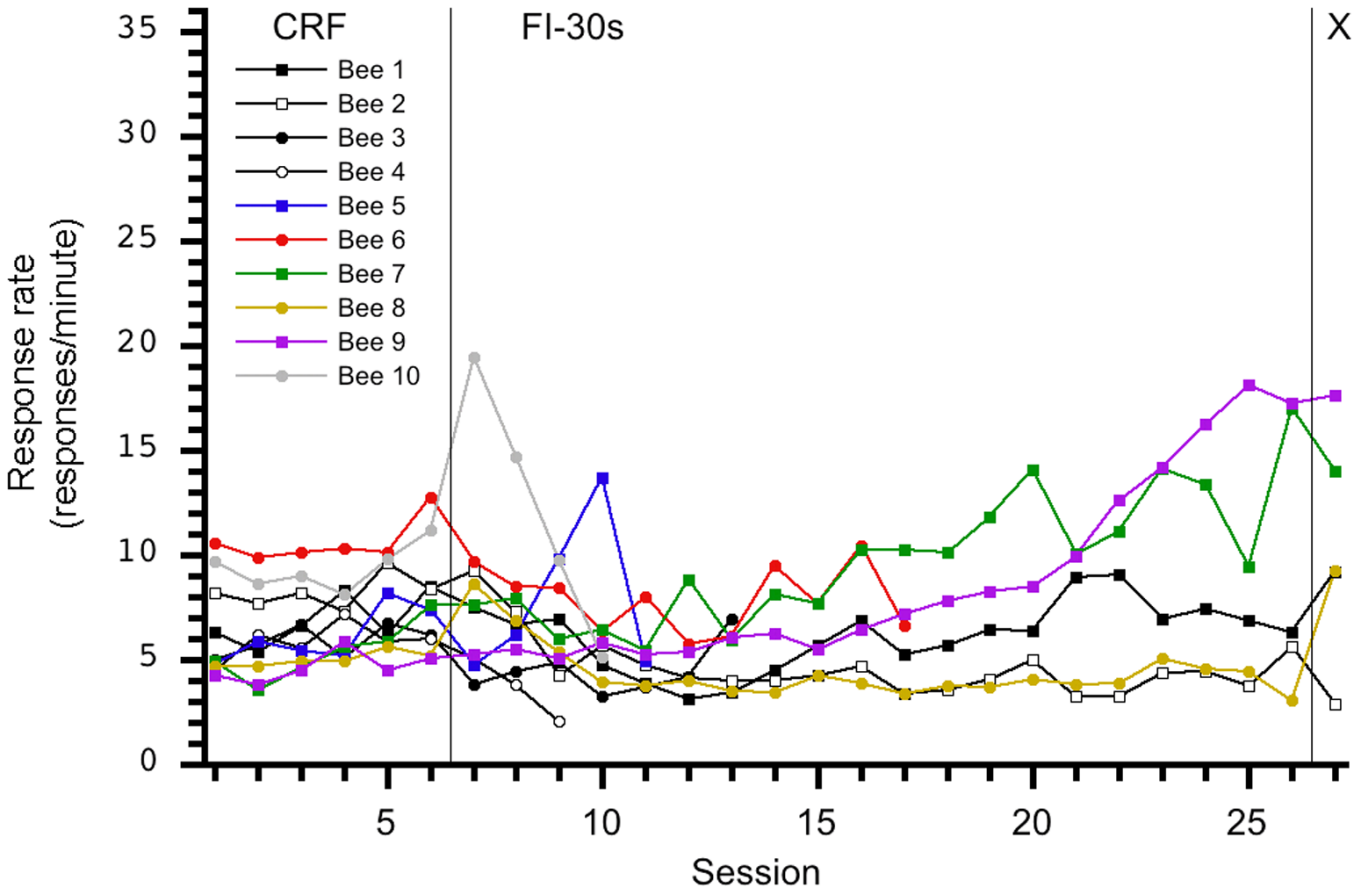
Individual response rates per session for the 0-30-X group. An increase in response rate is not observed during the transition from CRF to FI-30s. However, as FI-30s sessions continued, some individuals increase their response rates per session while others maintain uniform responding.

**Figure 5:**
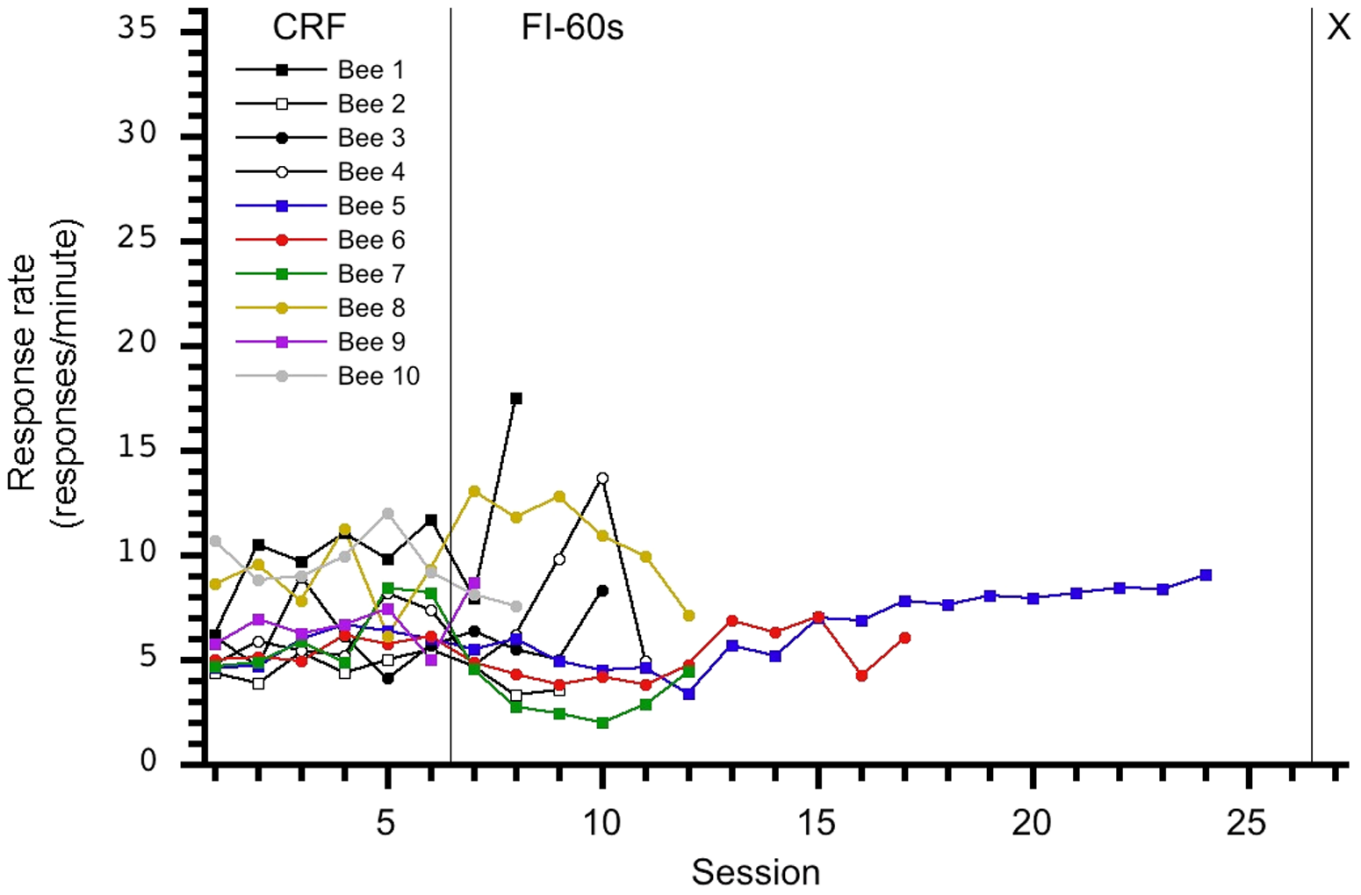
Individual response rates per session for the 0-60-X group. An increase in response rate is not observed during the transition from CRF to FI-60s; indeed, a decrease in response rate is observed for some subjects.

**Figure 6:**
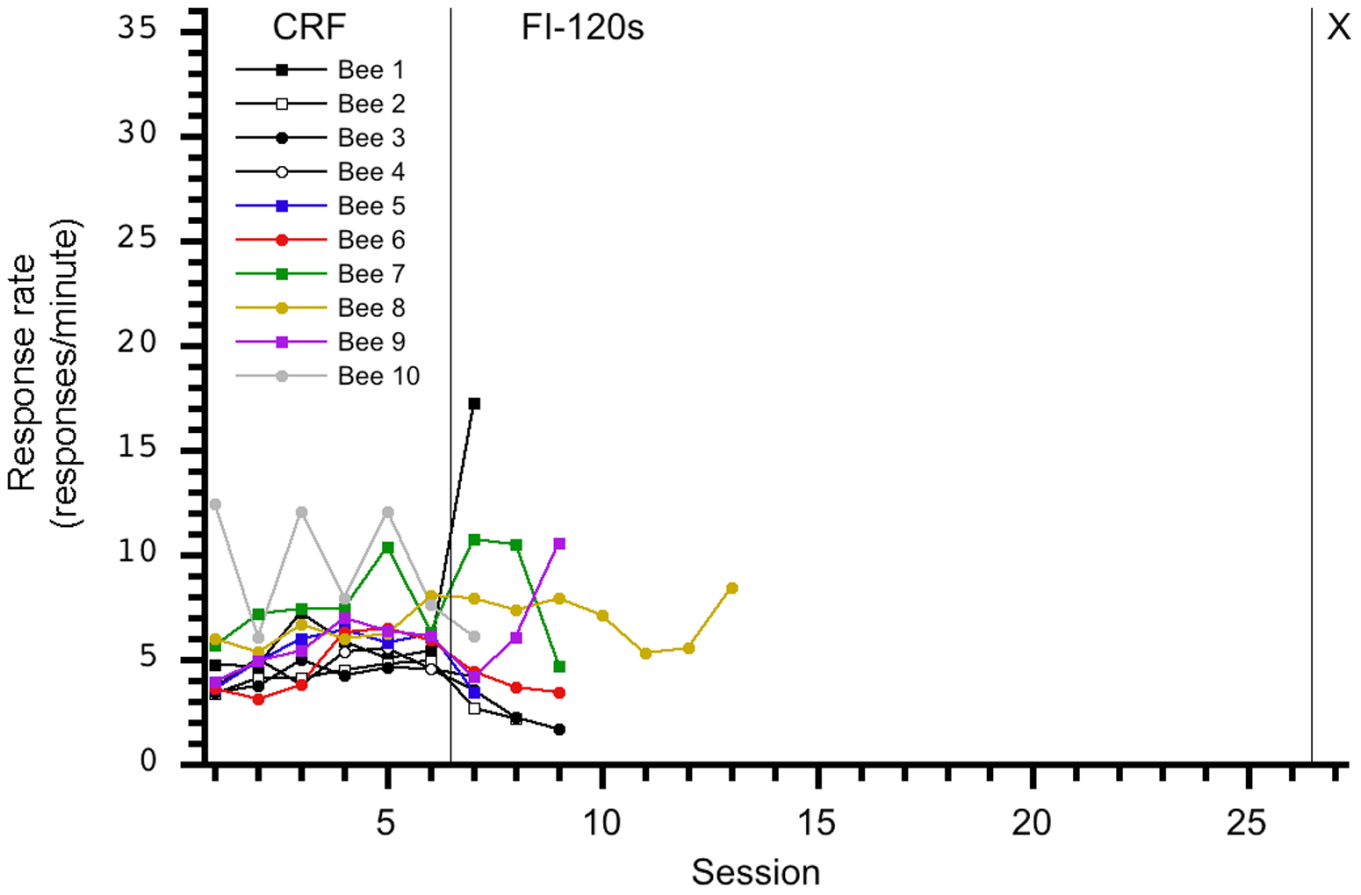
Individual response rates per session for the 0-120-X group. An increase in response rate is not observed during the transition from CRF to FI-120s.

Our initial findings did not support evidence of temporal control in this sample of honey bees. Figure 1 displays each group's session average number of responses and indicates response rates increased across FI sessions. This finding contrasts with reports [13] of the decreasing rate of response across sessions during the transition from CRF to FI but may indicate subjects learned to tolerate or habituate to the delays of reinforcement. Inspection of the individuals' performance contained in Figures 2, 3, 4, 5, and 6 depict this aggregate effect might be driven by a few individual's response rates and accompanying drop-outs of lower responding individuals and also supports the utility of an individual analysis.

Figure 1 also displays, for each group, what session subjects ceased responding or returning to the apparatus. All ten control 0-0-X subjects completed the experiment while eight 0-15-X subjects and five 0-30-X subjects completed the experiment. No 0-60-X and 0-120-X subjects maintained responding for 20 FI sessions; this drop-out effect indicates our subjects could not tolerate FI schedules longer than one minute. Moreover, drop-out percentages during shorter FI schedules reiterate the need for an individual analysis rather than an aggregate group analysis as multiple patterns of responding occurred in each group. Finally, Figure 1 reveals responding was maintained and relatively stable for subjects that completed the experiment.

### Response Rate

An increase in response rate as reinforcement availability approaches has been suggested to indicate temporal control [12], [44]. To perform this assessment, responses were chunked into two equal-duration bins for each fixed interval as doing so addresses the question: do subjects make more responses towards the end of the interval compared to the beginning? For example, an FI 30-sec would be divided into two 15-sec bins and the number of responses occurring during the first 15-sec bin would be compared with the number of responses occurring during the last 15-sec bin. If temporal control was present, the dichotomy between the bins should be clear. In order to compare individual response rates between the two bins within each interval, we used Observation Oriented Modeling (OOM) to compute an observed proportion correct classification (PCC) value between our observed data and a two-order *a priori* prediction. Based on previous FI literature [44], we predicted response rates would be greater as the schedule requirement approached. A PCC value comparing the instances of the observed data matching the ordinal prediction divided by the total number of comparisons was computed. Finally, each individual's observed response rate data underwent 100 randomizations and produced a range of PCC value outcomes of these randomizations. This range of PCC values obtained from the randomizations was compared to the PCC value of the observed data to produce a chance-value (*c*-value). To create group data, we pooled all individuals within a given FI schedule to obtain group comparison data without relying on aggregate summary statistics.

**Figure 7:**
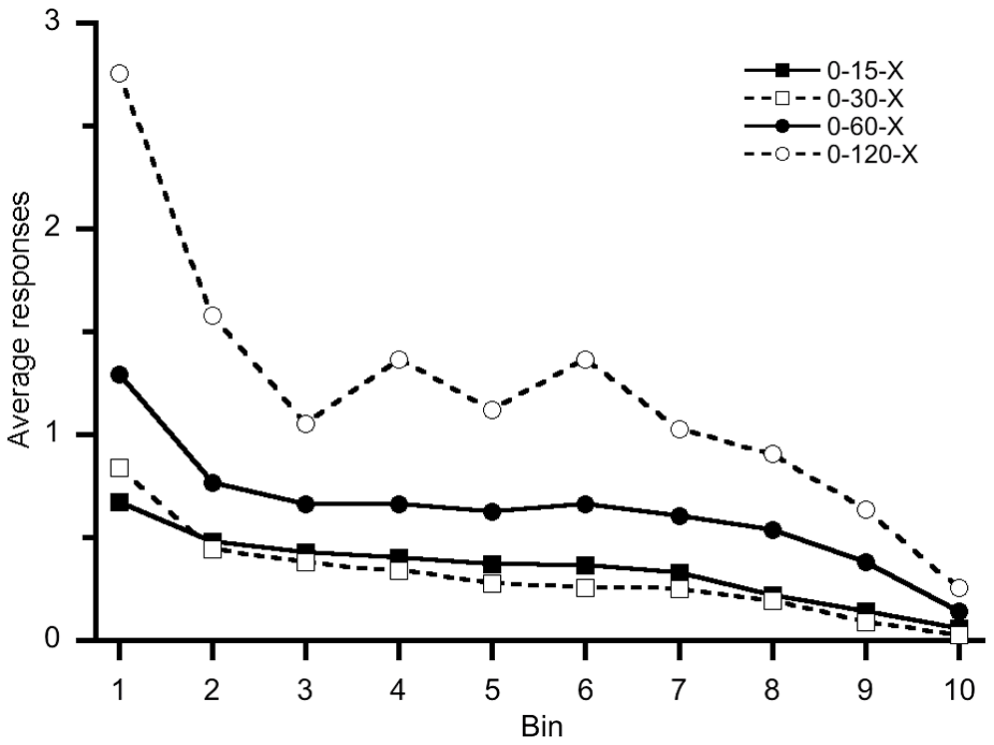
Group average number of hole-entering responses across ten bins when including all FI trials and sessions. A general downward trend is observed for all groups. The first bin, on average, contains the highest number of responses, and the last bin, on average, contains the lowest number of responses for all groups. This ten bin graphical representation resembles more of an extinction curve rather than a traditional FI “scallop” or “break-and-run” pattern of responding.

Our ordinal two-bin response rate analysis did not produce evidence of temporal control. Table 1 displays PCC values and accompanying *c*-values for each individual bee and pooled group response rate ordinal assessments while assessing every interval during the FI schedule condition. An inspection of Table 1 reveals no individual bees made more responses later in the interval than in the beginning of the interval when considering every FI trial and session as all PCC values were below 0.50 and all *c*-values were above an arbitrary 0.05 criteria. While our ordinal analysis was performed on two bins, we graphically represented response rate patterns using ten bins (Figure 7) to assess any potential artifacts related to our two-bin analysis. To create Figure 7, we first divided every FI trial into ten equal duration bins and then calculated each bin's average response rate when considering all FI trials from every subject in each group. A near perfect monotonically decreasing trend is observed across ten bins thus suggesting a lack of temporal control. Additionally, Figure 7 reveals the first and last bins contain the averages' highest and lowest number of responses, respectively. To facilitate comparison with existing and future findings, Table 2 displays group averages and standard deviations of response rates per minute; no systematic aggregate trends were observed.

**Table 1:** Hole-entering response rate ordinal comparisons within groups and individuals under the prediction responding would increase across the interval.

		Group	Bee 1	Bee 2	Bee 3	Bee 4	Bee 5	Bee 6	Bee 7	Bee 8	Bee 9	Bee 10
**0-15-X**	**PCC**	0.11	0.10	0.07	0.28	0.13	0.08	0.05	0.07	0.08	0.08	0.16
	***c*** **-value**	1.00	1.00	1.00	1.00	1.00	1.00	1.00	1.00	1.00	1.00	1.00
**0-30-X**	**PCC**	0.20	0.06	0.14	0.03	0.00	0.20	0.10	0.26	0.34	0.27	0.08
	***c*** **-value**	1.00	1.00	1.00	1.00	1.00	0.97	1.00	1.00	0.93	1.00	1.00
**0-60-X**	**PCC**	0.22	0.22	0.12	0.00	0.20	0.31	0.25	0.11	0.07	0.00	0.00
	***c*** **-value**	1.00	0.98	1.00	1.00	0.96	1.00	1.00	1.00	1.00	1.00	1.00
**0-120-X**	**PCC**	0.19	0.00	0.00	0.13	0.00	0.00	0.30	0.00	0.26	0.14	0.00
	***c*** **-value**	1.00	1.00	1.00	1.00	1.00	1.00	0.98	1.00	0.99	1.00	1.00

Intervals were divided into two equal duration bins, and these bins were ordinally compared. PCC values and accompanying *c*-values are displayed for individuals and pooled groups. No subjects responded at a higher frequency later in the interval (highest PCC value was 0.34; lowest *c*-value was 0.93).

**Table 2:** Group average and standard deviations of hole-entering response rates for baseline (BL) and FI conditions.

Group	BL Mean	FI Mean	Mean Difference	BL SD	FI SD	SD Difference
0-0-X	6.89	8.46	1.57	2.46	2.51	0.05
0-15-X	6.22	10.36	4.14	1.77	6.02	4.24
0-30-X	6.82	7.00	0.18	2.11	3.63	1.52
0-60-X	6.88	6.66	−0.22	2.23	3.04	0.80
0-120-X	5.76	6.08	0.32	2.05	3.53	1.48

Response rates are expressed in responses per minute. Baseline group averages imply roughly equal groups prior to the condition change. The 0-15-X group's standard deviation (SD) indicates non-homogeneous data during the FI condition.

We also assessed if subjects responded at higher levels later in the fixed interval as subjects experienced more FI sessions compared to earlier in the experiment by ordinally comparing the first and last FI sessions' PCC values. Using the same ordinal comparison method previously described, we predicted that higher response rate bin PCC values would be observed later in the experiment. We assessed this prediction by comparing the last FI session's two-bin response rate comparison PCC value with the first session's two-bin response rate comparison PCC value. We did observe temporal control improvement in responding across sessions (pooled PCC value: 0.72; pooled *c*-value: <0.01). However, even though 72% of the responses in the FI final session matched the ordinal prediction more than responding during the first FI session, no individuals displayed convincing improvement. Table 3 displays individual subject's PCC values and accompanying *c*-values for the final FI session bin comparison; no individual's final session's PCC value differed from randomized uniformity.

**Table 3:** Hole-entering response rate ordinal comparisons within individuals for the final FI session under the prediction that responding would increase across the interval.

		Bee 1	Bee 2	Bee 3	Bee 4	Bee 5	Bee 6	Bee 7	Bee 8	Bee 9	Bee 10
0-15-X	PCC	*	0.00	0.3	*	0.17	0.00	0.38	0.11	0.00	0.25
	*c*-value	*	1.00	0.92	*	0.97	1.00	0.78	1.00	1.00	0.88
0-30-X	PCC	0.10	0.33	*	*	*	*	0.43	0.50	0.64	*
	*c*-value	0.98	0.80	*	*	*	*	0.62	0.14	0.17	*

Intervals were divided into two equal duration bins, and combinations of these bins were ordinally compared. PCC values and accompanying *c*-values are displayed for individuals. Only one subject's PCC value was higher than a binomial comparison, but did not reliably differ from the randomized PCC values.

^*^Subject did not complete the 20^th^ FI session.

### Post Reinforcement Pause (PRP)

An increase in PRP at longer FI schedules has been suggested to indicate temporal control [12], [44]. To perform this assessment, we compared PRP between baseline and FI schedule conditions. Using the same ordinal analysis method previously described, we compared every baseline PRP to every PRP during the FI schedule condition under the prediction that baseline PRPs would be shorter than the FI PRPs. Thus, instead of comparing pairs of bins for each interval, we compared combinations of all baseline PRPs and FI PRPs for each individual bee. Table 4 displays PCC values and accompanying *c*-values for individual bees and their pooled groups for every interval during the FI schedule condition. When considering pooled group assessments, all FI groups produced PCC values at or above 0.5 seemingly indicating a marginal group effect. However, individual bees drastically varied in their “wait-times” on FI schedules compared to baseline performance. Fifteen out of forty subjects scored below a PCC value of 0.5 while ten subjects scored above a PCC value of 0.7. Table 5 displays group averages and standard deviations of group PRP durations and reveals an impressive aggregated group PRP mean difference for only one experimental group.

**Table 4:** PRP ordinal comparisons within groups and individuals under the prediction longer PRPs would be observed during the FI trials compared to baseline trials.

		Group	Bee 1	Bee 2	Bee 3	Bee 4	Bee 5	Bee 6	Bee 7	Bee 8	Bee 9	Bee 10
**0-0-X**	**PCC**	0.44	0.59	0.55	0.29	0.33	0.72	0.50	0.38	0.44	0.43	0.21
	***c*** **-value**	1.00	0.01	0.01	1.00	1.00	1.00	0.03	1.00	1.00	1.00	1.00
**0-15-X**	**PCC**	0.56	0.71	0.75	0.52	0.74	0.64	0.32	0.60	0.38	0.38	0.62
	***c*** **-value**	0.01	0.01	0.01	0.01	0.01	0.01	1.00	0.01	1.00	1.00	0.01
**0-30-X**	**PCC**	0.60	0.48	0.74	0.50	0.50	0.57	0.60	0.82	0.84	0.37	0.71
	***c*** **-value**	0.01	0.99	0.01	0.52	0.57	0.01	0.01	0.01	0.01	1.00	0.01
**0-60-X**	**PCC**	0.52	0.49	0.37	0.34	0.57	0.68	0.48	0.41	0.57	0.11	0.52
	***c*** **-value**	0.01	0.46	1.00	1.00	0.01	0.01	0.97	1.00	0.01	1.00	0.07
**0-120-X**	**PCC**	0.50	*	0.24	0.34	1.00	0.78	0.52	0.82	0.59	0.36	0.30
	**c-value**	0.02	*	1.00	1.00	0.01	0.01	0.04	0.13	0.01	1.00	1.00

PCC values and accompanying *c*-values are displayed for individuals and pooled groups. Ten of 40 subjects had larger PRPs during the FI trials compared to baseline for at least 70% of the observations (PCC values greater than 70%).

^*^Subject did not make enough responses for PRP analysis.

**Table 5:** Group average and standard deviations of PRP for baseline (BL) and FI conditions.

Group	BL Mean	FI Mean	Mean Difference	BL SD	FI SD	SD Difference
0-0-X	1.88	1.96	0.08	4.49	4.94	0.46
0-15-X	1.93	3.35	1.42	2.58	4.43	1.85
0-30-X	2.32	6.81	4.49	3.18	9.98	6.80
0-60-X	2.28	5.99	3.70	4.08	11.8	7.75
0-120-X	3.17	3.78	0.61	4.64	8.53	3.90

PRPS are expressed in seconds. Baseline group averages imply roughly equal groups prior to the condition change. The 0-15-X and 0-30-X groups' standard deviation (SD) indicates non-homogeneous data during the FI condition. An increase in group average PRP is observed.

### Analysis of “Scallop” Response Pattern

**Figure 8:**
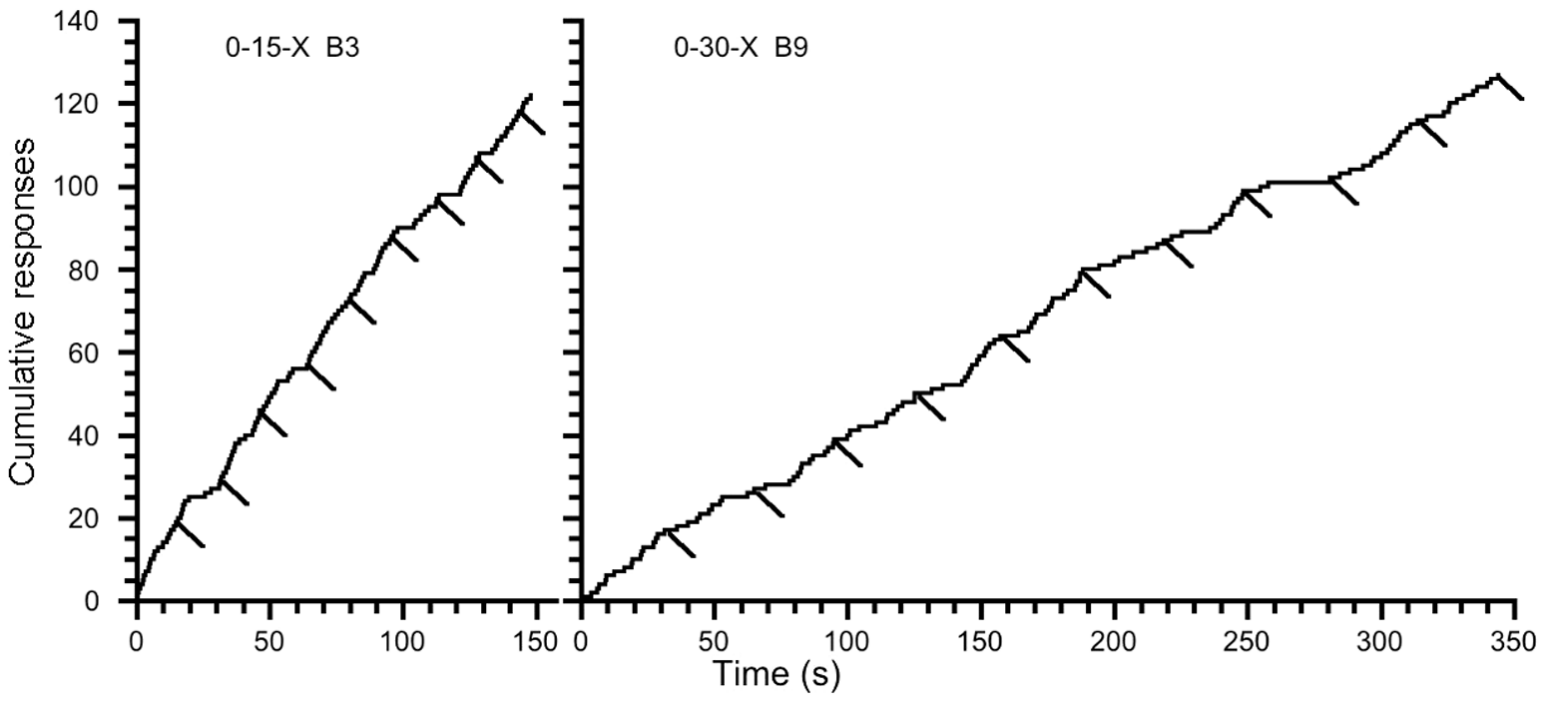
Cumulative hole-entering response records for the final session for Bee 3 in group 0-15-X (PCC value: 0.28) and Bee 9 in group 0-30-X (PCC value: 0.27). Response duration during reinforcement delivery is not displayed to illustrate a cumulative record of responses occurring between stimulus onset and a reinforced response. Responding resumes immediately following reinforcement consumption, and breaks in responding occurred intermittently during some trials. Short diagonal lines below the cumulative curve indicate reinforcement delivery.

Based on the individual analyses of response patterns (Table 1) and the reported observation of an increase in PCC values when comparing the final FI session to the first FI session, we selected a bee from the 0-15-X group and a bee from 0-30-X group with the best chances of displaying cumulative response records indicating temporal control. Figure 8 displays both the cumulative response record for the 26^th^ session for the subject with the highest ordinal two bin response comparison PCC value (0.28) in the 0-15-X group and the cumulative response record for the 26^th^ session for the subject with the second highest ordinal two bin response comparison PCC value (0.27) in the 0-30-X group. We selected the latter bee to represent the 0-30-X group because the subject with the highest PCC value (0.34) in the 0-30-X group made only 14 responses during the 20^th^ session and thus did not make a sufficient number of responses during its 20^th^ session to infer a response pattern. Neither selected subject produced “scalloped” or “break-and-run” cumulative response patterns.

### Extinction

Table 6 displays individual and group extinction response rates to indicate the variability of responding between and within groups. To further assess the effects of exposure to FI schedules, we performed a three-way ordinal comparison of each group's individual's extinction response rate utilizing the same ordinal analysis method we used to assess PRP. Based on the findings reported in [21], we predicted bees exposed to higher FI schedules would have a higher response rate in extinction than subjects only reinforced on CRF. However, we only included the 0-0-X, 0-15-X, and 0-30-X groups in this comparison as no subjects in the 0-60-X and 0-120-X groups completed 20 sessions of their respective FI schedules (hence the three-way comparison); subjects reinforced on longer schedules dropped-out and did not undergo the extinction session. Thus, we ordinally compared combinations of groups' individual's response rates during extinction following the ordinal prediction: 0-0-X <0–15-X <0–30-X. In addition to an observed PCC value and *c*-value, this three-way condition ordinal pattern prediction also provides a very sensitive complete PCC value and *c*-value. Extinction response rates were higher for subjects exposed to a FI schedule compared to control CRF schedule subjects (observed PCC value: 0.67, observed *c*-value: <0.01; complete PCC value: 0.34, *c*-value: <0.01). However, extinction response rates for subjects exposed to the FI 15-sec schedule were not smaller than response rates for subjects exposed to the FI 30-sec schedule (PCC value: 0.55; *c*-value: 0.32).

**Table 6:** Individual hole-entering responses per minute during the extinction condition.

	Group	Bee 1	Bee 2	Bee 3	Bee 4	Bee 5	Bee 6	Bee 7	Bee 8	Bee 9	Bee 10
**0-0-X**	6.37	2.2	2.0	4.9	15.3	2.5	8.8	9.9	4.0	10.4	3.7
**0-15-X**	9.125	*	5.8	19.4	*	5.2	12.4	5.2	6.3	6.8	11.9
**0-30-X**	10.12	9.4	2.9	*	*	*	*	11.2	8.9	18.2	*
**0-60-X**	N/A	*	*	*	*	*	*	*	*	*	*
**0-120-X**	N/A	*	*	*	*	*	*	*	*	*	*

Extinction response rates were higher for subjects exposed to a FI schedule compared to control CRF schedule subjects (observed PCC value: 0.67, observed *c*-value: <0.01; complete PCC value: 0.34, *c*-value: <0.01). However, extinction response rates for subjects exposed to the FI 15-sec schedule were not smaller than response rates for subjects exposed to the FI 30-sec schedule (PCC value: 0.55; *c*-value: 0.32).

^*^Subject stopped returning before the extinction condition.

## Discussion

We did not produce traditionally accepted evidence of temporal control in our sample of honey bees when considering the present individual analyses. Responding did not increase as the FI requirements approached and there was no systematic increase in PRP compared to baseline responding; indeed, we observed the opposite response pattern that would be predicted based on traditional vertebrate fixed interval investigations (Figure 7). While we observed an improvement in temporal control across sessions by comparing the first and last session's PCC values for response rate, no individual's final FI trials approximated evidence of temporal control. An individual increase in PRP was observed when subjects moved from CRF to an FI schedule, but these latencies did not approximate the durations reported in vertebrate literature [45].

When considering group assessments using pooled individuals, PCC values greater than 60% were not observed for the PRP analysis. Only 25% of subjects exposed to an FI schedule produced longer PRPs compared to previous baseline performance. Our two main findings of a relatively uniform response rate patterns across the fixed interval and an only marginal increase in PRP when subject responding was reinforced on an FI schedule are echoed in our individual cumulative curve analysis. Finally, reinforcing a subject's responding on FI 60-sec or FI 120-sec schedules resulted in the subject not returning to the apparatus for the full 20 sessions of the FI condition. Drop-out percentages for subjects with responding reinforced on an FI 15-sec and FI 30-sec also indicate some of our subjects did not tolerate the delays of intermittent reinforcement inherent in an FI schedule.

[20] describes increases in response rate towards the end of the interval as a ‘rudimentary’ form of temporal control (p. 93), so we primarily sought to investigate if honey bees can meet one of the most basic criteria of temporal control. We also investigated PRP to compare our results with the findings of [22]. Following [55], our findings lend another example demonstrating the independence of PRP and response rate and support the opinion [44] that multiple measures must be used to assess temporal control on FI schedules.

Our findings are in accordance with both previous FI investigations in invertebrates even though [21] and [22] reported seemingly contrasting findings. [21] did not find any evidence of temporal control in honey bees using a qualitative analysis of cumulative curves. In the cumulative curves reported in [21], PRPs were not identifiable and no increase in response rate within the interval was reported. Our cumulative curves reported in Figure 8 echo [21]. Additionally, our extinction response rate assessment confirmed [21]; bees previously exposed to only a CRF schedule made fewer responses compared to bees previously exposed to a FI schedule during extinction. [22] reported group average differences in PRP and we observed pooled group data produced longer PRPs when responding was reinforced on an FI schedule compared to a CRF schedule. Table 5 also reveals a mean difference between baseline and FI PRPs for our shortest FI experimental group. However, our method of individual subject analyses indicates these group averages in PRP were not followed by 37.5% of subjects and raises the possibility the group PRP effect reported in Table 5 and the group PRP effect reported in [22] may be due to an unrepresentative, abstract aggregate analysis.

[22] is the only publication supporting temporal control in an invertebrate. However, [22] did not perform an assessment of response rate, or perform an individual subject analysis; therefore an unequivocal interpretation of temporal control is not conclusive. Moreover, [44] explained why multiple measurements are necessary to assess temporal control on FI schedules; [54] labeled PRP as a more variable measure; and [56] observed PRP was a more sensitive and variable measure than response rate. Simply stated, PRP is a more liberal assessment of temporal control and cannot be fully conceptualized without considering response rate. Thus, we cannot gauge if temporal control has been demonstrated in an invertebrate especially when there is no evidence of “scalloped” or “break-and-run” cumulative response pattern.

In contrast to circadian rhythms, interval timing is far from being ubiquitous in vertebrates; thus, interval timing may be a more difficult task than circadian rhythms [20], [24], [56], [57]. Indeed, the existence of interval timing may be rare in invertebrate species given the differences in central nervous system complexity. Alternatively, [58] suggests that although honey bees show many of the behavioral abilities of vertebrates, honey bees may not be using the same neural mechanisms as vertebrates as observed in responses to aversive stimuli and stimulus removal [59]-[61]. Indeed, [23] indicates interval timing can be accomplished via several different approaches. Molecular studies have linked interval timing to elements of circadian rhythms; however, these elements appear to be distinct cellular mechanisms [62]. This finding suggests that circadian rhythms may be an indicator of rudimentary interval timing in honey bees and perhaps insects in general. [62] is consistent with the two prior inharmonious reports in invertebrate species as well as the results of the present experiment.

Honey bee circadian timing may be far less precise than posited by [63]. The *en masse* arrival of foragers when floral resources become productive daily [28], [29] was thought to be a result solely of circadian rhythms [64]. However, a limited number of bees monitor a site at any time; when the foraging site becomes productive, the monitoring bees alert the colony via the nectar's odor [65], [66]. Indeed, simply injecting a puff of the scent associated with the nectar into the hive elicits substantial re-recruitment of foragers to the flower patch in what [67] labels ‘*scent-triggered navigation*’ [68], [69]. Furthermore, monitoring occurs all day long for a site offering nectar rewards for a specific 2-hour period in the afternoon [70]. This finding contradicts what is expected from precision circadian rhythm triggered foraging even with behavior influenced by ‘expectation’ of reward.

While honey bees and bumble bees exhibit similar foraging and life history patterns, bumble bees appear to have surprisingly different neural mechanisms compared to honey bees. Research over the last decade highlights substantial differences in foraging behavior between these related bee genera [71]–[73]. Furthermore, bumble bees do not exhibit the blue-yellow type of flower constancy observed in honey bees [71], [74]. Additionally, bumble bees are less flower type constant and often visit several flower species in one foraging bout [72], [75]. While both species are rudimentary compared to vertebrates, bumble bees may have more sophisticated mechanisms for temporal control compared to honey bees; however, further investigations are required to substantiate this claim.

A final consideration concerns the effect of relative food quality on speed of trophallaxis (i.e. unloading sucrose at the hive); it is possible our subjects experienced a decrease in perceived quality of the foraging location due to the interreinforcement delays caused by the fixed intervals [76], [77]. However, as we did measure perceived quality of food via proxy of trophallactic rates, we cannot conclude whether the effects reported in [76], [77] could have impacted the measured intersession intervals and thus may have affected the observed drop-out rates of the present data.

## Conclusions

The present investigation utilized a non-aggregate based data analysis method to assess PRP and response rates when honey bee responding was reinforced on fixed interval schedules. No individual subject produced evidence of interval timing when assessing both response rate and PRP. While we did not produce evidence of timing behaviors in honey bees when responses are reinforced on fixed interval schedules, this lack of evidence contributes to a growing body of work that cautions the use of anthropomorphic assumptions when concerning invertebrate learning; understanding what species are or are not able to perform complex behaviors is critical from a comparative perspective [78]. The reported findings connect the divergence between previous invertebrate temporal investigations and cautions against drawing conclusions of timing when only analyzing group data. Future investigations should include individual analyses of multiple measures as reported here before making species and biological comparisons. Moreover, comparative work must contend with the different responses between investigations (i.e. head-poke versus proboscis extension). While the free-flying honey bee behavioral fixed interval model may be useful for ecological investigations of both interval timing and circadian rhythms, other interval procedures such as a choice protocol reported in [79] may be useful for ethologist and behaviorist method comparisons of interval timing.

## Supporting Information

File S1 **0-0-X Raw Data.** Contains all ten 0-0-X subjects' cleaned data as well as group analyses of intersession interval, reinforcers per visit, interresponse times, response duration, visit duration, response rate, and post reinforcement pause.
(XLSX)

File S2 **0-15-X Raw Data.** Contains all ten 0-15-X subjects' cleaned data as well as group analyses of intersession interval, reinforcers per visit, responses per reinforce per visit, interresponse times, response duration, visit duration, response rate, and post reinforcement pause.
(XLSX)

File S3 **0-30-X Raw Data.** Contains all ten 0-30-X subjects' cleaned data as well as group analyses of intersession interval, reinforcers per visit, responses per reinforce per visit, interresponse times, response duration, visit duration, response rate, and post reinforcement pause.
(XLSX)

File S4 **0-60-X Raw Data.** Contains all ten 0-60-X subjects' cleaned data as well as group analyses of intersession interval, reinforcers per visit, responses per reinforce per visit, interresponse times, response duration, visit duration, response rate, and post reinforcement pause.
(XLSX)

File S5 **0-120-X Raw Data.** Contains all ten 0-120-X subjects' cleaned data as well as group analyses of intersession interval, reinforcers per visit, responses per reinforce per visit, interresponse times, response duration, visit duration, response rate, and post reinforcement pause.
(XLSX)

File S6 **Response Rate Data.** Contains all 50 subjects' cleaned response rate data for between-group comparisons.
(XLSX)

File S7 **Post Reinforcement Pause Data.** Contains all 50 subjects' cleaned post reinforcement pause data for between-group comparisons.
(XLSX)
